# Nutritional Status and Associated Factors among Primary Schoolchildren from Pastoral Communities, Mieso-Mulu District, Sitti Zone, Somali Regional State, Eastern Ethiopia: Institution-Based Cross-Sectional Study

**DOI:** 10.1155/2021/6630620

**Published:** 2021-09-16

**Authors:** Alayou Geletaw, Gudina Egata, Fitsum Weldegebreal, Gesessew Kibr, Mohammed Semaw

**Affiliations:** ^1^Somalia Regional Health Bureau, Jigjiga, Ethiopia; ^2^School of Public Health, College of Health and Medical Sciences, Haramaya University, Harar, Ethiopia; ^3^Department of Public Health Nutrition and Dietetics, College of Health Sciences, Addis Ababa University, Addis Ababa, Ethiopia; ^4^Department of Medical Laboratory Sciences, College of Health and Medical Sciences, Haramaya University, Harar, Ethiopia; ^5^Department of Food and Nutritional Sciences, Faculty of Agriculture, Shambu Campus, Wollega University, Shambu, Ethiopia

## Abstract

**Background:**

Child undernourishment is the disturbance of body function arising from a dietary imbalance between body demand and supply, which is the most serious public health problem in developing countries.

**Objective:**

This study aimed to assess the magnitude of nutritional status and associated factors among full-cycle primary schoolchildren in pastoral communities in the Mieso-Mulu district, Sitti Zone, Somali Regional State of Ethiopia.

**Methods:**

An institution-based cross-sectional study design was used. Study participants were selected using two-stage sampling procedures. Data were collected using structured, translated, pretested, and interviewer-administered questionnaires. The weight and height were measured using a calibrated digital scale and a Seca Rod stadiometer, respectively. Microscopic identification of intestinal parasites was done. Multicollinearity was checked for independent variables. Height for age *z* scores (HAZ) and body mass index for age *z* scores (BAZ) were used to determine the nutritional status of children. Logistic regression with both bivariate analysis and multivariate analysis was applied to identify associated factors with the nutritional status of children. Adjusted odds ratios were reported and the level of statistical significance was declared at a *P* value <0.05.

**Results:**

The magnitudes of thinness and stunting were 13.1% [95% CI: 10.6%, 15.7%] and 24.6% [95% CI: 21.3%, 27.9%], respectively. Being male, not using a bed net, and the presence of intestinal parasitic infection were among the factors associated with thinness. Family size of less than five, household food insecurity, and unavailability of the latrine were among the factors associated with stunting.

**Conclusion:**

This study revealed that stunting and thinness are major health problems among schoolchildren. Household food insecurity, intestinal parasitic infection, bed net utilization, and the availability of latrine were some of the major factors significantly associated with undernutrition. Local policymakers, health programmers, nutritionists, health practitioners, and nongovernmental organizations should enhance the nutritional status of schoolchildren by using information dissemination interventions, particularly in improving waste disposal, sanitation/hygiene, latrine facilities, and school-based deworming. Furthermore, awareness creation using nutrition promotion and encouraging communities to attempt to diversify locally available and low-cost nutritionally effective food items to improve food consumption and distribution within a household is recommended to reduce the prevalence of undernutrition among schoolchildren.

## 1. Introduction

Malnutrition is one of the most challenging and complex global problems affecting development, particularly that of the privileged and the poor. Schoolchildren are vulnerable to undernutrition due to low social status, poor diet, ill health, and inappropriate care [1]. Nearly 690 million people were undernourished globally in 2019, with 144 million children stunted (21.3%) and 47 million wasting (6.9%) [2, 3]. In 2018, 5.3 million children died before reaching their fifth birthday, many of them as a result of malnutrition [3]. The highest is found in Africa south of the Sahara and in South Asia. With 2020 global hunger index values of 27.8 and 26.0, respectively, Africa south of the Sahara and South Asia have the greatest levels of serious hunger and undernutrition among the world regions [3]. 32.7% of children in Africa south of the Sahara and 33.2% of children in South Asia were underweight for their age, indicating chronic malnutrition [3, 4].

Furthermore, stunting affects 20 to 80% of school-aged children [5, 6]. Malnutrition is responsible for more than half of all child fatalities in developing countries [2, 4]. Various driving factors have been identified to be associated with undernutrition among children, such as globalization, urbanization, inequities, crises, health epidemics, and humanitarian emergencies, which lead to inadequate nutrient intake, inadequate care, and infectious diseases [2, 7–9]. Infections like intestinal parasitosis are related to increased morbidity due to reduced appetite, malabsorption, and gastrointestinal blood loss causing undernutrition with consequent impairment of growth and development of children. Schoolchildren carry the heaviest burden of parasite-associated morbidity due to their habits of playing or handling infested soil, eating with soiled hands, unhygienic toilet practices, drinking and eating contaminated water/food, and sharing toys, bedding, and clothing [8, 9]. Undernutrition increases the frequency and severity of infections in children, making them more likely to die from them [3, 10].

In Ethiopia, the recent 2019 Mini Ethiopian Demographic and Health Survey indicated that 37% of children were stunted, 7% wasted, and 21% underweight, respectively [11]. Besides, different findings reported undernutrition among schoolchildren [12–18]. According to the report of the 2018 Ethiopia nutrition profile, about 27% of children in the Somali region were stunted, 29% underweight, and 23% wasted, respectively [19]. Furthermore, the undernutrition of school-aged children was studied in the Somali Regional State of Ethiopia [20–22]. Nutritional deprivation of schoolchildren can limit the full development of physical and cognitive potential, resulting in weight loss, low school enrolment, recurrent illness, high absenteeism, early dropout, and unsatisfactory academic performance [8–10, 23, 24]. Food security, safe water, good sanitation facilities, hygiene practices, maternal care practices, access to health services [21], and cultural and environmental habits and practices [25] can all affect children's overall nutritional status. Stunting has been linked to factors such as age, gender, family size, lack of use of a bed net, and sickness [17, 21, 24, 26, 27]. Meanwhile, family size, drinking water, latrine availability, household income index, handwashing with soap after using the toilet, diarrheal disease, and child food insecurity were all linked to thinness [21, 27–32].

The government of Ethiopia has been working to promote sustainable and high-quality school health and nutrition interventions [33]. Additionally, to improve children's nutritional status, the government is implementing several strategies, including the 2004 National Strategy for Infant and Young Child Feeding Practices, the 2005/2006 National Nutrition Strategy, and the 2008 National Nutrition Program, with the help of various stakeholders [34]. As a result, throughout the last few decades, the country has made promising progress in addressing child malnutrition. Even though the country's undernutrition situation has improved, it remains one of the countries with the worst rates of malnutrition in the world, posing a substantial barrier to good child health outcomes [11]. Pastoral communities are hard to reach population groups to address health and nutrition services as required due to their migratory lifestyle and unfavorable environmental conditions based solely on herding [35]. Schoolchildren in pastoral communities are affected by undernutrition, especially due to unsanitary environmental conditions [36, 37].

Despite a lot of breakthroughs in health and nutrition services in developing countries, such as Ethiopia, the nutritional status of schoolchildren is not often included in health and nutrition surveys, and there is no current comprehensive review of their nutritional status [38, 39]. The pastoral and agropastoral areas were also disregarded in previous studies on children's nutritional conditions in Ethiopia [28, 30, 40–42]. Another driving force behind this research was a dearth of data on schoolchildren's health, as well as governments' and aid agencies' rising interest in the link between health and education. The Somali Regional State, which is primarily populated by pastoralist populations, has been classified as one of the country's hotspot regions, with severe food insecurity, increased child malnutrition rates, and recurring droughts. Investigation of the problem and identification of its causal variables within this framework, according to [43], are a key step in developing effective methods to ameliorate the problem. In particular, in the Mieso-Mulu district, the burden of undernutrition and factors related to undernutrition among primary schoolchildren have not yet been studied to design appropriate low-cost health and nutrition interventions. Therefore, this study aimed to assess the magnitude of undernutrition and associated factors among primary schoolchildren in pastoral communities in the Mieso-Mulu district, Sitti Zone, Somali Regional State, Eastern Ethiopia.

## 2. Materials and Methods

### 2.1. Study Area and Period

**Figure 1:**
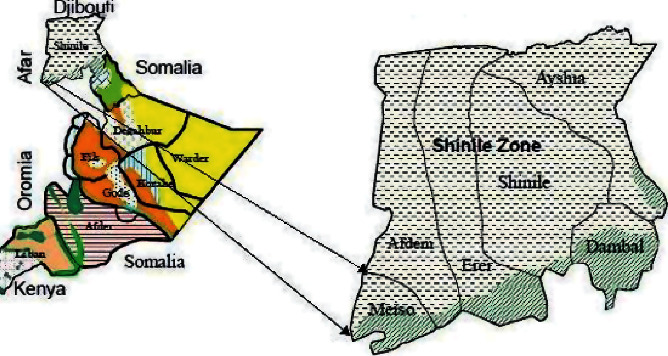
Maps of the study area (source: Save the Children, 2007).

This study was conducted in the Mieso-Mulu district, Sitti Zone, Somali Regional State, Ethiopia. The district is located about 327 km from the capital of the region, Jigjiga, and 312 km from the capital of the country, Addis Ababa, respectively. The Mieso-Mulu district is found in the southwest of the Sitti Zone (formerly known as Shinile) and is bordered by the Afar Region to the northwest, the Oromia region to the south, and Afdem Woreda to the east (see Figure 1). The district has five primary schools [44], which serve 3,143 (1,767 males and 1,376 females) school children. The study was conducted from March to April 2019.

### 2.2. Study Design and Population

An institution-based quantitative cross-sectional study design with descriptive and analytical statistics was used. The source population consisted of all children attending full-cycle primary schools in the Mieso-Mulu district and their respective parents or guardians. The study population consisted of all children attending the selected three full-cycle primary schools in the Mieso-Mulu district and their parents or guardians. Primary schoolchildren who had a history of treatment for intestinal parasitic infection two weeks before the study period and physical deformities (of legs and/or spine) were excluded.

### 2.3. Sample Size Determination and Sampling Procedure

The sample size required for the study was determined using Epi Info version 7 StatCalc by considering latrine availability, determined in the previous study as an important predictor of stunting (45.7%) among schoolchildren who had no latrine and 32.2% of those who had a latrine [15], the two-sided confidence level of 95%, the margin of error of 5%, and power of 80%. For each variable, a design effect of 1.5% and a nonresponse rate of 5% were considered to get the minimum acceptable sample size. Therefore, the final sample size was 690, of which 671 study subjects participated. A representative sample of schoolchildren was selected by using a multistage sampling technique. In the first stage, a simple random sampling technique was used to select three schools from the total of five full-cycle primary schools found in the district. Allocation of the sample of students to the selected schools and grade levels was performed proportionally based on the number of students in each school and grade level, respectively. Study participants were selected from each grade level by a systematic sampling technique. The sampling interval was calculated by using the 2018/2019 academic year class rosters as a sampling frame. Therefore, by dividing the total number of students in each grade level by the number of sampled students in the respective class, the sampling interval (*k*) was two. A random number was selected from one or two by the lottery system. The randomly picked number was one. Finally, children with odd numbers were selected until they got the required sample size from each grade. Accordingly, about 315, 230, and 145 schoolchildren were selected and included from the schools of Afase, Gedugas, and Somedaye, respectively.

### 2.4. Data Collection Methods and Instruments

#### 2.4.1. Sociodemographic, Household, and Dietary Information

Sociodemographic, household, and dietary related data were collected using structured, interviewer-administered, and pretested questionnaires. Measurement tools for sociodemographic, household, and dietary related data were adapted from different pieces of literature [45–48]. Household food insecurity status was assessed using the nine Household Food Insecurity Access Scale (HFIAS) questions [49]. The age of the children was collected by asking the parents and by cross-checking the school records. Children were screened at schools and parents or guardians of the selected children were traced back for a face-to-face interview. Before actual data collection, a pretest was done in Muli primary school by taking 5% of the total sample size on 35 participants before the actual data collection period. The simplicity, flow, and consistency of the questionnaires were checked. The reliability of the questionnaire was checked (Cronbach's alpha = 0.71). About six nurse professionals were assigned to administer the interview and three laboratory professionals were recruited to collect and examine stool samples. Two B.S. degree health officers were assigned to supervise the activity along with the principal investigator.

#### 2.4.2. Anthropometric Measurements

Anthropometric data of the selected schoolchildren was obtained at schools by using standardized techniques and calibrated equipment. The weight was measured using a calibrated digital scale (Seca, designed in Germany and made in China, max: 150 kg) to the nearest 0.1 kg and the height was recorded to the nearest 0.1 cm by using a Seca Rod stadiometer with a sliding bar. Measurement of height was carried out, while the heels, calves, buttocks, scapulae, and heads were against the measuring rod in a standing position with the arms hanging freely. Their heads were positioned in the Frankfort horizontal plane. A sliding bar was lowered until it touched the head of the child [50]. All measurements were taken with children wearing their school uniform and barefoot.

The quality of measurements was ensured by first having an expert measure ten children. Following that, data collectors measured the weight and height of the same children twice, with a break between the first and second measurements. The relative technical errors of measurements for inter- and intraexaminers were compared with cut-off points. The calculated relative measurement of each data collector was within the acceptable limits (less than 1.5% and 1%, respectively). For further minimization of errors during anthropometric measurements, each data collector took two consecutive measurements of each child and an average of the two measurements was used to calculate the anthropometric indices.

#### 2.4.3. Parasitological Examination

For parasitological examination, proper instructions were given to the study subjects to protect the sample from contamination with materials like soil, urine, and water. They were provided a clean wooden applicator stick and a clean, dry, and leak-proof plastic cup labeled with the subject's serial number and instructed to bring 2 grams (about thumb size) of the fresh stool sample. Laboratory professionals have checked reagents, specimen collection, and processed types of equipment. Specimen preparation and examination were done according to the recommended standard operating procedures [51]. On-site examination of specimens was done within 30 minutes of sample collection using a direct wet mount technique to detect helminths (eggs and/or larvae) and protozoa (cysts and/or trophozoites). To increase the parasite detection rate, the remaining samples were preserved in a tube containing 10 milliliters of 10% formalin and transported to the Mieso-Mulu health center for microscopic examination after processing using the formalin-ether sedimentation concentration technique as described in the World Health Organization (WHO) guideline [52]. Two laboratory professionals examined each stool sample and a senior laboratory professional checked the consistency of the results.

### 2.5. Operational Definitions

  Undernutrition. Schoolchildren with a *z* score value of less than −2 standard deviation (−2SD) for height for age (stunting) and body mass index for age (thinness) with the WHO median reference population value were considered as undernourished.
  Intestinal parasites. They are organisms that live in or take nourishment from another organism in the intestinal tract, including both helminths and protozoa.
  Household food insecurity. A household that worries about not having enough food sometimes or often and/or is unable to eat preferred foods is mildly food-insecure. Households that sacrifice quality more frequently, by eating a monotonous diet or undesirable foods sometimes or often, are moderately food-insecure. Households that have been forced to cut back on meal size or the number of meals often and/or experience any of the three most common eating occasions are severely food-insecure.
  Full-cycle primary school. It is a school that comprises grades from one to eight.
  Schoolchildren. In this study, schoolchildren were students who attended classes from grade one to grade eight.

### 2.6. Quality Control

The questionnaire was prepared in English, translated into the local language (Somaligna), and back-translated into English by different experts to maintain consistency. To assure data quality, training was given for two days to data collectors by the principal investigator on the objective of the study, the relevance of the study, confidentiality of information, respondent's rights, informed consent, methods of the interview, skills for taking anthropometric measurements, and stool collection technique. During data collection, the supervisor and investigator did close supervision. The completeness and consistency of questionnaires were reviewed and ensured daily. Data editing and coding were done before entry. To maintain the quality and consistency of the data, double data entry was done by using the computer software EpiData version 3.1.

### 2.7. Data Processing and Analysis

Data was exported from EpiData version 3.1 (the EpiData Association, Odense, Denmark, Europe) to Statistical Package for the Social Sciences version 20 software (Armonk, NY, USA) for data cleaning and analysis. The data were sorted, summarized, cleaned, and checked for missing values using frequencies and cross-tabulations. HAZ and BAZ were determined based on WHO 2007 AnthroPlus software. Children who fall below −2SD and −3SD from the median of the reference population were regarded as moderately and severely malnourished, respectively [50]. Thinness and stunting were dichotomized as “yes = 1” and “no = 0.” Schoolchildren with a *z* score < −2SD were coded as “1” and those with a *z* score > −2SD were coded as “0.” Independent variables were also coded based on previous related studies and the distribution of responses in the data.

During analysis, descriptive statistics were used for an overall description of the result. Multicollinearity between covariates was checked using the variance of the inflation factor. Candidate variables with 95% CI and a *P* value <0.25 during bivariate analysis were selected and transformed into multivariable analysis to control the effects of confounding variables and to determine the independent predictors of nutritional status. The odds ratio along with a 95% confidence interval was reported to assess the association between predictor variables and the nutritional status of children. A *P* value <0.05 was declared as statistically significant. The fitness of the model was tested by the Hosmer-Lemeshow goodness-of-fit test for stunting (*P* value = 0.39) and thinness (*P* value = 0.26).

## 3. Results and Discussion

### 3.1. Sociodemographic and Household Characteristics

In the current study, 671 children attending full-cycle primary schools and their parents or guardians participated with a response rate of 97%. The age of the children ranged from 6 to 17 years, with a mean (±SD) age of 10.9 ± 2.67 years. Moreover, more than half (55.9%) of households were food-insecure during the study period. Out of 671 households, 52.8% of them managed domestic waste on an open field, 24.4% in a pit, and 22.8% by burning it, respectively. About 279 (41.6%) households have not treated their drinking water and around 31% of households have no latrine. 236 (35.2%) households did not use a bed net while sleeping (see Table 1).

### 3.2. Dietary Patterns of Schoolchildren

Of 671 schoolchildren, only 123 (18.3%) children had more than three meals per day. However, the remaining 548 (81.7%) schoolchildren had almost three meals per day.

### 3.3. Intestinal Parasitic Infections among Schoolchildren

**Figure 2:**
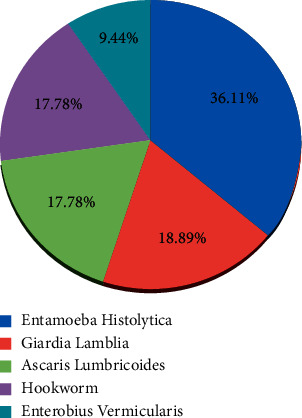
Proportion of isolated intestinal parasite species among infected children attending primary schools in pastoral communities, Mieso-Mulu district, Sitti Zone, Eastern Ethiopia, 2019.

Out of 671 primary schoolchildren, 180 (26.8%) children were infected by intestinal parasites, 14.7% by protozoa, and the remaining 12.1% by helminths. Among the identified parasitic species, the most dominant isolated parasites were *Entamoeba histolytica* (36.11%), followed by *Giardia lamblia* (18.89%), *Ascaris lumbricoides* (17.78%), Hookworm (17.78%), and *Enterobius vermicularis* (9.44%), respectively (see Figure 2).

### 3.4. Nutritional Status of Schoolchildren

**Figure 3:**
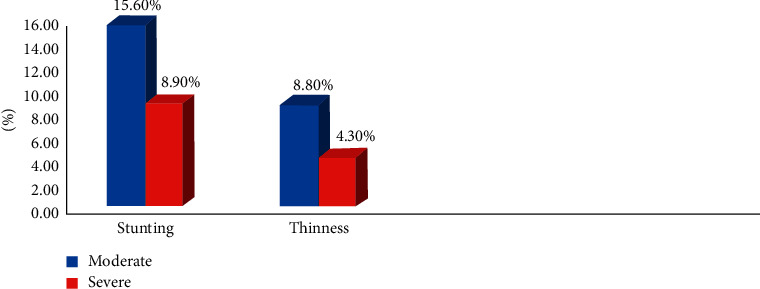
Magnitudes of stunting and thinness among children attending full-cycle primary schools in pastoral communities, Mieso-Mulu district, Sitti Zone, Somali Regional State, Eastern Ethiopia, 2019.

In this study, the magnitudes of thinness and stunting among schoolchildren were 13.1% (95% CI: 10.6%, 15.7%) and 24.6% (95% CI: 21.3%, 27.9%), respectively (see Figure 3). The magnitude of thinness is comparable with study results reported from Dale woreda (14%) [13], Harari (8.7%) [18], Somali refugee camps (15.2%) [22], Nigeria's Akwa Ibom State (10.9%) [53], Tanzania (11.3%) [54], and Babile town (15.7%) [55]. On the other hand, the magnitude of thinness in this study was lower than study results in the Philippines (27.8%) [56]. Similarly, the magnitude observed in schoolchildren from pastoralist and agropastoralist communities (22.9%) [21], Fogera district (37.2%) [47], Bahir Dar district (26.7%) [57], and Meket district (37.5%) [58] was lower. This variation might be due to the differences in the availability of health education programs for schoolchildren. The possible reason for this discrepancy might be due to the higher magnitude of intestinal parasitic infections among primary schoolchildren in those study areas. The overall magnitude of intestinal parasitic infection in primary schoolchildren from the study done in Bahir Dar district, Ethiopia, was 52.4% [57], which was much higher than the magnitude in the current study (26.8%). It is documented that intestinal parasitic infection can lead to undernutrition due to competition for essential nutrients as well as endogenous nutrient losses [57].

Furthermore, the magnitude of stunting among primary schoolchildren was 24.6%. This finding is in agreement with studies reported on the national figures of stunting in Ethiopia (23%) [7], Dale woreda (25.6%) [13], Southern Ethiopia (28%) [14], Uganda's Wakiso District (22.5%) [59], and Jimma Zone (24.1%) [60]. Unlike the present study, a lower magnitude of stunting was reported from Ethiopia, such as Southern Ethiopia (28%), Harari (15.8%), Bahir Dar city (15.13%), Babile town (11.2%), Bahir Dar district (18.3%), and Addis Ababa (19.6%) [14, 18, 55, 57, 61, 62], respectively. In contrast, the results are lower than the findings from Gondar town (46.1%) [17], Arba Minch city (41.9%) [24], Humbo district (57%) [48], and Mecha district (37.9%) [63]. This discrepancy might be associated with the difference in socioeconomic and household characteristics of study participants. In the current study, around 56% of households were food-insecure. In addition, more than 50% of households dispose of domestic waste on an open field. These conditions might increase the magnitude of stunting by affecting the type and frequency of children's food and exposing them to frequent illnesses. The magnitude of stunting was higher in some other study results reported from different parts of Ethiopia, such as the Gurage zone of Enemorena-Ener district (39%) [15], Fogera and Libo Kemekem districts (rural 42.7% and urban 29.2%) [26], and Fogera district (30.7%) [47]. This disagreement might be due to the difference in household facilities, the livelihood of the communities, and study settings. In the Gurage zone, Enemorena-Ener district, more than 50% of households had no latrine, which might contribute to the highest magnitude of stunting in this district [15]. In the Fogera and Libo Kemekem districts, Herrador et al. (2014) reported a higher magnitude of stunting than that in the current study by using a community-based cross-sectional study [26].

### 3.5. Predicting Factors of Nutritional Status of Schoolchildren

To determine factors associated with the nutritional status of schoolchildren, a logistic regression model with both bivariate analysis and multivariate analysis was applied. Accordingly, sociodemographic variables, household characteristics, dietary information, and parasitic infection were considered as independent variables and nutritional status (thinness and stunting) was considered as a dependent variable.

#### 3.5.1. Factors Associated with Thinness

From bivariate analysis, age, sex, site of waste disposal, bed net utilization, and presence of intestinal parasitic infection were significantly associated with thinness at a *P* value less than 0.05. After adjusting for all candidate variables in the multivariable analysis, sex, site of waste disposal, bed net utilization, and intestinal parasitic infection remained statistically significant at a *P* value less than 0.05 (see Table 2). Accordingly, male children were 1.86 times [AOR = 1.86; 95% CI: 1.12, 3.09] more likely to be thin than their counterparts, which is consistent with other study results reported in Ethiopia [29, 32, 58]. Boys have less body fat and more muscle mass compared to girls; thus, they have a higher energy demand and burn more calories compared to girls. This could be explained in part by the fact that boys in the same age groups are more vulnerable to health inequities than their female counterparts [20].

Many Ethiopian studies, including those in Dollo Ado district [20], Bule Hora district [64], and Benna Tsemay district [65], found comparable outcomes. Male children were shown to be more sensitive to malnutrition than female children in systematic studies conducted in Ethiopia and other developing countries [1,66], owing to differences in eating frequency, energy expenditure, and exposure to health problems. This conclusion differs from that in a study of school-aged street youths conducted in Southwest Ethiopia [27]. Primary schoolchildren from families who manage their domestic waste by dumping it into the pit or by burning it were 50% [AOR = 0.50; 95% CI: 0.26, 0.96] and 63% [AOR = 0.37; 95% CI: 0.17, 0.91] less likely to be thin than primary schoolchildren from families who dispose of it in an open field. This could be due to the reason that improper waste management might increase the occurrence of frequent infectious diseases which lead to undernutrition through increasing caloric needs [67].

Regarding bed net utilization, children from families that did not use bed nets for malaria control while sleeping were 4.22 times [AOR = 4.22; 95% CI: 2.52, 7.03] more likely to be thin than their counterparts. Accordingly, the finding can be explained by the fact that malaria has a well-known impact on children's wellbeing in the form of anorexia, diarrhea, vomiting, fever, and increased consumption of protein to fight infection [68], all of which lead to body development failure and weight loss. In addition, children who were affected by intestinal parasitic infection were 2.25 times [AOR = 2.25; 95% CI: 1.36, 3.73] more likely to be thin than children who had no intestinal parasitic infection. This finding is in agreement with reports from Ethiopia from Enemorena-Ener district, National data, Wolaita Sodo town, and Bahir Dar district [15, 29, 32, 57]. Intestinal parasitism can cause or aggravate undernutrition through impaired nutrient absorption, reduced appetite, and competition for the existing nutrients that leads to acute undernutrition. Moreover, intestinal parasitic infections can cause vomiting, diarrhea, anorexia, abdominal pain, and nausea that may result in reduced food intake, thereby further reducing nutrient availability [53, 57].

#### 3.5.2. Factors Associated with Stunting

From bivariate analysis, age, availability of latrine, site of waste disposal, and household food security status were significantly associated with stunting at a *P* value less than 0.05. In multivariable analysis, age, family size, availability of latrine, site of waste disposal, and household food security status were significantly associated with stunting at a *P* value less than 0.05 (see Table 3). Accordingly, 14–17-year-old children were 48% less [AOR = 0.52; 95% CI: 0.29, 0.95] likely to be stunted than 6–9-year-old children. Numerous other studies revealed that the likelihood of stunting was higher as the child got older [13, 18, 20, 27, 29, 31, 32, 45, 53, 54, 59, 61]. However, in this study, children aged 14–17 years were less likely to be stunted than children aged 6–9 years. This could be due to adequate nutrient intake in addition to higher requirements for older schoolchildren, or it could be that conditions are improving over time, and older kids are more likely than younger kids to have healthy eating habits. Younger children have practices of playing in infested soil, eating without washing their hands, drinking, and eating polluted water and food [8, 9, 57], all of which allow harmful microorganisms to be introduced. Due to diminished appetite, malabsorption, and gastrointestinal blood loss, such patterns in children result in undernutrition, impairing body growth and development. Reports indicate that the percentage of children involved in cattle herding decreases as age increases. As mentioned before, keeping animals for a longer period might expose them to different zoonotic diseases, poor food intake, and isolation, which potentially leads to stunting [69].

Children with a family size of less than or equal to five were 2.14 times [AOR = 2.14; 95% CI: 1.20, 3.81] more likely to be stunted than children with a family size greater than eight. However, other studies conducted in Ethiopia, such as Mieso Woreda of the Somali region [21], Fogera district [26], Mecha district [45], Hambo district [48], and Addis Ababa [62], revealed that the likelihood of stunting was higher among children of large families. Due to the scarcity of human power, children from smaller family-sized households may have a high opportunity to engage in different activities in their communities, such as herding and consistently caring for domestic animals, especially staying out of the house for an extended period with animals in search of water and grazing land. The Food and Agriculture Organization reported that, in pastoral communities, child labor is simply part of traditional life and economic organization. In particular, caring for animals for a longer period might expose children to inadequate dietary intake, isolation, lack of appetite, fatigue, and infections related to animals (zoonotic diseases), which potentially lead to undernutrition [69].

Children from families without a latrine were 2.21 times [AOR = 2.21; 95% CI: 1.48, 3.29] more likely to be stunted than those who have a latrine. Regarding the site of waste disposal, families who disposed of domestic waste in a pit were 64% less [AOR = 0.36; 95% CI: 0.21, 0.63] likely to have stunted children than families who disposed of domestic waste in an open field. Similar findings were reported from the Gurage zone in the Enemorena-Ener district [15]. In addition, children from families that dump domestic waste in a pit were less likely to be stunted than those who dispose of it in an open field. This finding is similar to a study done in the southern Ethiopian Humbo district [48]. This may be due to improper sanitation that serves as a vehicle for intestinal parasites/bacteria that would increase the risk of infection, especially diarrheal disease, resulting in depletion of micronutrients. Moreover, children from food-insecure households were 2.79 times [AOR = 2.79; 95% CI: 1.81, 4.31] more likely to be stunted than children from food-secure households. This finding is in agreement with other studies conducted in Mieso Woreda, Jimma Zone, Wolaita Sodo town, Tehuledere district, and Dale district [13, 21, 30–32], respectively. This might be because children in food-insecure households face reduced dietary variety and intake of food, which will later result in nutrition-related problems.

This study is one of the few studies revealing the magnitude of stunting and thinness and their predictor variables among primary schoolchildren in pastoral communities. Further community-based studies are necessary to include children who might not be enrolled in school. Nevertheless, it is not free from the following limitations. Due to the cross-sectional nature of this study, it was not possible to establish a causal relationship between dependent and independent variables. A single 24-hour recall of dietary data might not reflect the usual intake of children, and caretakers' recall of the previous day's intake did not include foods consumed outside the home. The exclusion of children with physical deformities of the limbs and/or spine might have resulted in some extent of selection bias for conditions that are associated with nutritional deficiencies in children. Information about the age of the child was obtained by asking parents. This may incur recall bias and it may have some impact on the estimates of anthropometric indicators. However, efforts were made to cross-check the school records.

## 4. Conclusions and Recommendations

This study revealed that stunting and thinness are moderate and serious public health problems for schoolchildren, respectively. Being male, not using a bed net, the presence of intestinal parasitic infection, and domestic waste disposal by either pit or burning independently predicted the occurrence of thinness among full-cycle primary schoolchildren. In addition, stunting was significantly associated with age of 14–17 years, family size ≤5, unavailability of a latrine, domestic waste disposal in a pit, and household food insecurity. Based on this finding, the district health office needs to monitor and evaluate the implementation of health extension packages related to disease prevention and control, environmental sanitation, and personal hygiene to reduce the impact of mosquito bites and unhygienic personal and environmental conditions on the health and growth of schoolchildren. There is a need for the implementation of school-based deworming programs in collaboration with schools, education officials, local nongovernmental organizations, and other concerned bodies to reduce parasite burdens to prevent potentially adverse effects on the nutritional and health status of schoolchildren. The district administration officials need to take initiatives to improve the overall socioeconomic situation and household food security status of the community in collaboration with higher hierarchy officials to reduce the menace of undernutrition in the community. Besides this, to reduce the prevalence of undernutrition among schoolchildren, raising awareness through nutrition education/promotion and motivating communities to seek diversifying commonly accessible and low-cost nutritionally appropriate types of food to enhance eating habits and distribution inside a household is highly recommended. Moreover, more large-scale studies or national nutritional surveys need to consider schoolchildren as one component to regularly assess the nutritional status of this age group.

## Figures and Tables

**Table 1:** Sociodemographic and household characteristics of children attending primary schools in pastoral communities, Mieso-Mulu district, Sitti Zone, Somali Regional State, Eastern Ethiopia, 2019.

Variables	Category	Frequency	Percentage (%)
Sex	Male	345	51.4
	Female	326	48.6

Age in years	6–9	216	32.2
	10–13	322	48
	14–17	133	19.8

Mothers' education	Illiterate	551	82.1
	Literate	120	17.9

Mothers' occupation	Housewife	472	70.3
	Working mothers^∗^	199	29.7

Occupation of fathers	Pastoral	267	39.8
	Merchant	196	29.2
	Employer	99	14.8
	Others^∗∗^	109	16.2

Family size	<5	236	32.5
	6–8	321	47.8
	>8	114	17

Treatment of drinking water	Yes	392	58.4
	No	279	41.6

Site of waste disposal	Open field	354	52.8
	Pit	164	24.4
	Burn	153	22.8

Availability of latrine	Yes	461	68.7
	No	210	31.3

Use of bed net	Yes	435	64.8
	No	236	35.2

Household food security	Yes	296	44.1
	No	375	55.9

^∗^ **=** merchant/pastoral/employer; ^∗∗^  = daily laborers/no work.

**Table 2:** Factors associated with thinness among children attending primary schools in pastoral communities, Mieso-Mulu district, Sitti Zone, Somali Regional State, Eastern Ethiopia, 2019.

Variables		Thinness		COR (95% CI)	AOR (95% CI)
		Yes (%)	No (%)		
Age in years	6–9	19 (8.8%)	197 (91.2%)	1	1
	10–13	53 (16.5%)	269 (83.5%)	2.04 (1.17,3.56)^∗^	1.82 (0.99,3.31)
	14–17	16 (12%)	117 (88%)	1.42 (0.70, 2.87)	1.15 (0.53, 2.48)

Sex	Male	57 (16.5%)	288 (83.5%)	1.88 (1.18, 3.00)^∗^	1.86 (1.12, 3.09)^∗^
	Female	31 (13.1%)	295 (86.9%)	1	1

Mothers' education	Illiterate	77 (14%)	474 (86%)	1.61 (0.83, 3.13)	1.58 (0.68, 3.68)
	Literate	11 (9.2%)	109 (90.8%)	1	1

Mothers' occupation	Housewife	68 (14.4%)	404 (85.6%)	1.51 (0.89, 2.56)	1.10 (0.56, 2.17)
	Working mothers**^**	20 (10.1%)	179 (89.9%)	1	1

Fathers' occupation	Pastoral	33 (12.4%)	234 (87.6%)	0.82 (0.43, 1.60)	0.65 (0.31, 1.35)
	Merchant	23 (11.7%)	173 (88.3%)	0.77 (0.39, 1.52)	0.70 (0.32, 1.56)
	Employer	16 (16.2%)	83 (83.8%)	1.12 (0.53, 2.38)	1.56 (0.62, 3.94)
	Others**^^**	16 (16.7%)	93 (83.3%)	1	1

Family size	≤5	27 (11.4%)	209 (88.6%)	0.92 (0.46, 1.84)	1.42 (0.65, 3.06)
	6–8	47 (14.6%)	274 (85.4%)	1.23 (0.65, 2.32)	1.56 (0.79, 3.22)
	>8	14 (12.3%)	100 (87.7%)	1	1

Daily meal frequency	≤3	68 (12.4%)	480 (86.6%)	0.73 (0.42, 1.26)	0.64 (0.34, 1.19)
	>3	20 (16.3%)	103 (83.7%)	1	1

Water treatment	No	36 (12.9%)	243 (87.1%)	0.97 (0.61, 1.53)	
	Yes	52 (13.3%)	340 (86.7%)	1	

Latrine availability	No	28 (13.3%)	182 (86.7%)	1.03 (0.64, 1.66)	0.74 (0.42, 1.30)
	Yes	60 (13%)	401 (87%)	1	1

Waste disposal site	Open field	63 (17.8%)	291 (82.2%)	1	1
	Pit	15 (9.1%)	149 (90.9%)	0.47 (0.26, 0.85)^∗^	0.50 (0.26, 0.96)^∗^
	Burning	10 (6.5%)	143 (93.5%)	0.32 (0.16, 0.65)^∗^	0.37 (0.17, 0.91)^∗^

Bed net utilization	No	56 (23.7%)	180 (76.3%)	3.92 (2.45, 6.26)^∗∗^	4.22 (2.52, 7.03)^∗∗^
	Yes	32 (7.3%)	403 (92.6%)	1	1

HFIAS	Insecure	52 (13.9%)	323 (86.1%)	1.16 (0.74, 1.83)	1.07 (0.63, 1.84)
	Secure	36 (12.2%)	260 (87.8%)	1	1

Intestinal parasitic infection	Yes	38 (21.1%)	142 (78.9%)	2.36 (1.49, 3.75)^∗∗^	2.25 (1.36, 3.73)^∗∗^
	No	50 (10.2%)	441 (89.8%)	1	1

^∗^, *P* value < 0.05; ^∗∗^, *P* value < 0.01; ^^^merchant, pastoral, and employer; ^^^^daily laborer, no work; COR: crude odds ratio; AOR: adjusted odds ratio; CI: confidence interval.

**Table 3:** Factors associated with stunting among children attending primary schools in pastoral communities, Mieso-Mulu district, Sitti Zone, Somali Regional State, Eastern Ethiopia, 2019.

Variables		Stunting		COR (95% CI)	AOR (95% CI)
		Yes (%)	No (%)		
Age in years	6–9	65 (30.1%)	151 (69.9%)	1	1
	10–13	80 (28.8%)	242 (75.2%)	0.77 (0.52, 1.13)	0.91 (0.60, 1.38)
	14–17	20 (15%)	113 (85%)	0.41 (0.24, 0.72)^∗^	0.52 (0.29, 0.95)^∗^

Sex	Male	86 (24.9%)	259 (75.1%)	1.04 (0.73, 1.48)	
	Female	79 (24.2%)	247 (75.8%)	1	

Mothers' education	Illiterate	132 (24%)	419 (76%)	0.83 (0.53, 1.30)	
	Literate	33 (27.5%)	87 (72.5%)	1	

Mothers' occupation	Housewife	114 (24.2%)	358 (75.8%)	0.92 (0.63, 1.35)	
	Working mothers**^**	51 (25.6%)	148 (74.4%)	1	

Family size	≤5	71 (30.1%)	165 (69.9%)	1.61 (0.95, 2.74)	2.14 (1.20, 3.81)^∗^
	6–8	70 (21.8%)	251 (78.2%)	1.05 (0.62, 1.76)	1.11 (0.64, 1.93)
	>8	24 (21.1%)	90 (78.9%)	1	

Daily meal frequency	≤3	142 (25.9%)	406 (74.1%)	1.52 (0.930, 2.49)	0.88 (0.51, 1.54)
	>3	23 (21.7%)	100 (81.3%)	1	1

Fathers' occupation	Pastoral	58 (21.7%)	209 (78.3%)	0.80 (0.48, 1.35)	
	Merchant	55 (28.1%)	141 (71.9%)	1.13 (0.66, 1.92)	
	Employer	24 (24.2%)	75 (75.8%)	0.93 (0.49, 1.74)	
	Others**^^**	28 (25.7%)	81 (74.3%)	1	

Water treatment	Yes	93 (23.7%)	299 (76.3%)	1	
	No	72 (25.8%)	207 (74.2%)	1.12 (0.78, 1.60)	

Latrine availability	No	79 (37.6%)	131 (62.4%)	2.63 (1.83, 3.79)^∗∗^	2.21 (1.48, 3.29)^∗∗^
	Yes	86 (18.7%)	375 (81.3%)	1	1

Waste disposal site	Open field	108 (30.5%)	246 (69.5%)	1	1
	Pit	20 (12.2%)	144 (87.8%)	0.32 (0.19, 0.53)^∗∗^	0.36 (0.21, 0.62)^∗∗^
	Burning	37 (24.2%)	116 (75.8%)	0.73 (0.47, 1.12)	1.06 (0.65, 1.72)

Bed net utilization	No	54 (22.9%)	182 (77.1%)	0.87 (0.60, 1.26)	
	Yes	111 (25.5%)	324 (74.5%)	1	

HFIAS	Insecure	121 (32.3%)	254 (67.7%)	2.73 (1.85, 4.02)^∗∗^	2.79 (1.81, 4.31)^∗∗^
	Secure	44 (14.9%)	252 (85.1%)	1	1

Intestinal parasitic infection	Yes	39 (21.7%)	141 (78.3%)	0.80 (0.53, 1.21)	0.69 (0.44, 1.06)
	No	126 (25.7%)	365 (74.3%)	1	1

^∗^, *P* value < 0.05; ^∗∗^, *P* value < 0.01; ^^^merchant, pastoral, and employer; ^^^^daily laborer, no work; COR: crude odds ratio; AOR: adjusted odds ratio; CI: confidence interval.

## Data Availability

The datasets used and analyzed to support the findings of this study are available from the corresponding author upon reasonable request.

## Abbreviations

AOR: Adjusted odds ratio
BAZ: Body mass index for age *z* scores
BMI: Body mass index
COR: Crude odds ratio
CI: Confidence interval
HAZ: Height for age *z* scores
HFIAS: Household Food Insecurity Access Scale
SD: Standard deviation
WHO: World Health Organization.
